# Feasibility of Developing Radiotracers for MDM2: Synthesis and Preliminary Evaluation of an ^18^F-Labeled Analogue of the MDM2 Inhibitor SP-141

**DOI:** 10.3390/ph14040358

**Published:** 2021-04-13

**Authors:** Satish K. Chitneni, Zhengyuan Zhou, Brian E. Watts, Michael R. Zalutsky

**Affiliations:** 1 Department of Radiology, Duke University Medical Center, Durham, NC 27710, USA; zz89@duke.edu (Z.Z.); michael.zalutsky@duke.edu (M.R.Z.); 2 Duke Human Vaccine Institute, Duke University, Durham, NC 27708, USA; brian.watts@duke.edu

**Keywords:** MDM2, imaging, MDM2 inhibitor, fluorine-18, PET, radiotracer, SP-141, MCF-7, HepG2

## Abstract

Murine double minute 2 (MDM2), a negative regulator of the p53 tumor suppressor protein, is overexpressed in several human cancers. Herein we investigate the feasibility of developing ^18^F-labeled compounds based on the small molecule inhibitor SP-141 for imaging tumor MDM2 expression levels with positron emission tomography (PET). Three nonradioactive fluorinated SP-141 analogues, **1**–**3**, were synthesized, and their binding to the MDM2 protein was analyzed by surface plasmon resonance (SPR). One of these, a fluoroethoxy analogue, was labeled with fluorine-18 (^18^F) using ^18^F-fluorethyl bromide to provide [^18^F]**1** and evaluated in vitro and in vivo. SPR analysis confirmed the binding of the fluorinated analogues to MDM2 at 1.25–20 µM concentrations. Cell uptake studies revealed high uptake (67.5–71.4%/mg protein) and specificity of [^18^F]**1** in MCF7 and HepG2 cells. The uptake of [^18^F]**1** in these cells could be modulated using 100 µM SP-141, potentially reflecting changes in MDM2 expression because of p53 activation by SP-141. [^18^F]**1** exhibited stable uptake and retention in HepG2 tumor xenografts (~3 %ID/g) in vivo, but poor clearance from blood and other normal tissues, yielding low tumor-to-background ratios (<2) at 2 h post injection. Our results suggest that [^18^F]**1** has suboptimal characteristics for in vivo evaluation as a PET tracer for MDM2, but warrant radiolabeling and assessment of the other fluorinated analogues synthesized in this work, **2** and **3**, and potentially other molecular scaffolds for developing MDM2 targeted radiotracers.

## 1. Introduction

The tumor suppressor protein p53 plays a crucial role in maintaining the genomic stability of cells and preventing cancer formation [1]. The significance of p53 as a tumor suppressor protein is evident from the fact that nearly 50% of all human cancers carry mutations in the p53 gene (*TP53*), resulting in the loss of functional p53 protein in p53-mutant cancers. In tumors that have wild-type p53, it is often inactivated by the overexpression of its negative regulatory protein, murine double minute 2 (MDM2, or the human homolog HDM2) [2]. Under normal conditions, p53 levels are tightly regulated by the MDM2 protein, which engages in protein–protein interaction with p53 protein, targeting p53 for proteasomal degradation via its E3 ubiquitin ligase activity [3,4]. However, in response to cellular stresses, such as DNA damage, p53 levels are stabilized and induce transcriptional activation of specific genes that determine the outcome of the stress event—either repair and return to homeostasis or cell death [5,6]. Thus, MDM2 overexpression can inhibit the tumor suppressive function of p53 and favor continued proliferation and tumor cell survival [7]. Studies suggest that MDM2 is overexpressed in multiple human cancers [2], including breast cancer (15%), glioblastoma (14%), sarcomas (20%), and acute myeloid leukemia (AML), and has been shown to promote metastasis in some cancers [8,9,10,11]. Therefore, MDM2 is currently being investigated as a therapeutic target for the treatment of cancers that frequently overexpress MDM2 and as a means to reactivate the p53 pathway in wild-type p53 cancers. Both small molecules and peptide-based inhibitors of MDM2 are being pursued [12,13].

Since the first successful demonstration of MDM2 inhibition using the small molecule inhibitors Nutlin 1–3 by Vassilev et al. [14], several other molecular scaffolds have been identified as potential inhibitors of MDM2, including p53-based stapled peptides [12,15]. With regard to the mode of inhibition, most MDM2 inhibitors reported to date bind to the hydrophobic p53 binding pocket on the N-terminus of MDM2, blocking its interaction with the p53 protein. This allows p53 levels to rise and transcriptionally activate several p53-targeted genes, thereby facilitating DNA repair, cell cycle arrest, and apoptosis, among other functions [7]. To date, several small molecule inhibitors of MDM2 have been tested in clinical trials, including RG7112, RG7388, and AMG232. These studies demonstrated the ability of MDM2 inhibitors to activate the p53 pathway in tumor cells [13,16,17]. As expected, based on the mechanism of activity of MDM2 inhibitors, evidence from the aforementioned clinical studies suggests that the expression levels of MDM2 in tumor cells could serve as predictive and early response biomarker for the treatment of patients with MDM2-p53 targeted therapies [16]. 

**Figure 1:**
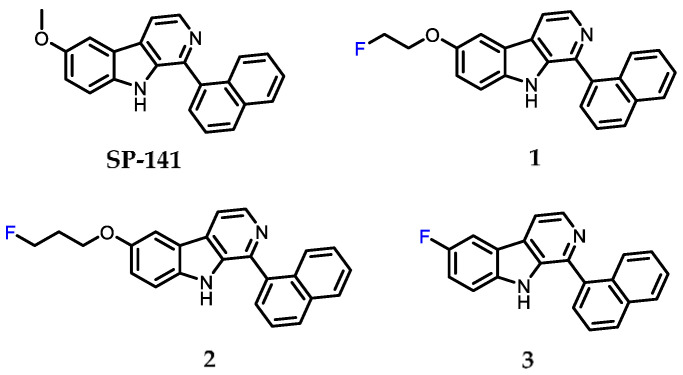
Chemical structure of the known MDM2 inhibitor SP-141, and the new fluorinated analogues (**1**–**3**) synthesized in the present work.

Currently, MDM2 gene amplification and/or protein overexpression in human cancers are assessed by ex vivo methods, such as fluorescence in-situ hybridization (FISH) and immunohistochemical (IHC) staining of tumor biopsy material [18,19]. Although these methods provide accurate information on MDM2 amplification/overexpression status, a noninvasive imaging method could facilitate real-time measurement of MDM2 expression levels in tumors by positron emission tomography (PET) imaging, including assessing treatment response in patients treated with MDM2 inhibitors. This provides a compelling rationale for the development of PET imaging agents for MDM2. To the best of our knowledge, there have been no published reports of radiotracer development for PET imaging of MDM2 at the protein level; however, Fu et al. demonstrated that a ^99m^Tc-labeled antisense oligonucleotide targeting MDM2 mRNA accumulated in MCF-7 tumor xenografts by single-photon emission computed tomography (SPECT) imaging, with a tumor-to-muscle ratio of ~4 at 10 h post injection [20,21]. However, delivery of oligonucleotides into the cells for binding to intracellular targets, such as MDM2, generally requires liposome encapsulation, as was the case for the aforementioned ^99m^Tc-labeled antisense oligonucleotide [20]. Given the ability of small molecules with appropriate lipophilicity to traverse the cell membrane more efficiently and the much higher abundance of protein molecules vs. mRNA in mammalian cells [22], small molecule MDM2 inhibitors are promising alternate candidates for developing radiotracers for the MDM2 protein [15,23]. Herein, we have investigated the feasibility of developing fluorine-18 (^18^F) labeled probes based on the chemical structure of the well-studied small molecule MDM2 inhibitor SP-141 (Figure 1) [24]. Studies suggest that SP-141 directly binds to the MDM2 protein and decreases its expression levels in tumor cells by promoting autoubiquitination and proteasomal degradation of MDM2 [24,25]. Additionally, SP-141 has shown promising preclinical activity against a variety of tumor types, including breast cancer, hepatocellular carcinoma, pancreatic cancer, and glioblastoma [24,25,26,27]. In view of these promising characteristics and its ease of synthesis, we have selected SP-141 as the first molecular scaffold for investigating the feasibility of developing ^18^F-labeled probes for MDM2 imaging with PET. Accordingly, we synthesized three fluorinated analogues of SP-141 by replacing the methoxy substituent on the β-carboline moiety with a fluoroethoxy, fluoropropoxy, or a fluorine function. The synthesized nonradioactive analogues were evaluated for their ability to bind to MDM2 by surface plasmon resonance (SPR) analysis. From these, a fluoroethoxy analogue of SP-141 was labeled with ^18^F and evaluated for its uptake in MDM2 expressing tumor cell lines in vitro. The tissue distribution and tumor uptake of the labeled compound were investigated in athymic mice bearing HepG2 hepatocellular carcinoma xenografts in vivo. 

## 2. Results

### 2.1. Chemistry

**Scheme 1:**
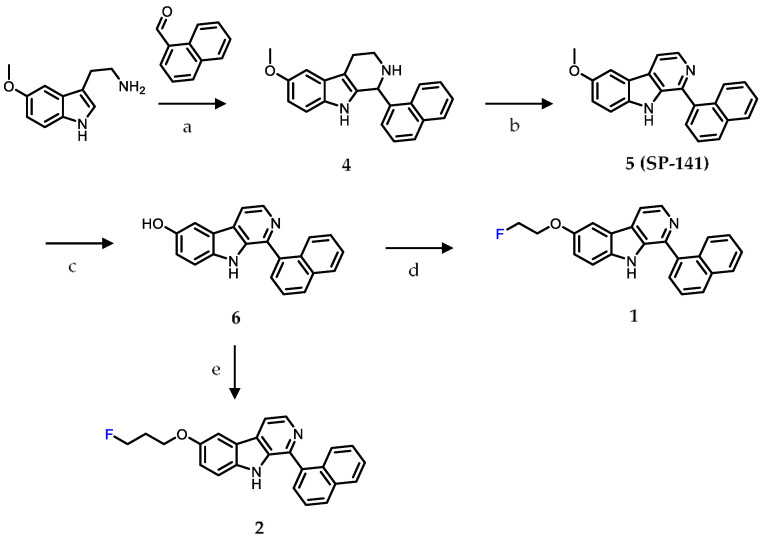
Synthesis scheme for SP-141 and the fluorinated analogues **1** and **2**. Reaction conditions: (**a**) Trifluoroacetic acid (TFA), 0 °C → room temperature (RT), tetrahydrofuran (THF), overnight; (**b**) Palladium on carbon (Pd/C), xylenes, reflux, 4 h; (**c**) Boron tribromide, dichloromethane, −70 °C → RT, RT, 3 d; (**d**) 2-Fluoroethyl bromide, cesium carbonate (Cs_2_CO_3_), dimethylformamide (DMF), 70 °C, 24 h; (**e**) 3-Fluoropropyl-4-methylbenzenesulfonate, Cs_2_CO_3_, methanol, 70 °C, 19 h.

SP-141 was selected as the initial model compound to investigate the feasibility of developing ^18^F-labeled agents for imaging MDM2 with PET. It has been reported that SP-141 binds to MDM2 with a binding affinity (*K*_i_) of 28 ± 6 nM, which is significantly higher than the binding affinity of the natural p53 peptide (1.1 ± 0.2 µM) for the MDM2 protein under the same conditions [24]. An additional consideration for selecting SP-141 for the design of radiotracers for MDM2 imaging is the possibility of substituting the methoxy group on the β-carboline moiety in SP-141 with a [^18^F]fluoroethoxy or [^18^F]fluoropropoxy function for ^18^F-labeling (Scheme 1). Based on the reported structure–activity relationship (SAR) data, this should have minimal impact on their affinity towards MDM2 [28]. In addition, the fluoro-β-carboline derivative **3**, where the 6-methoxy function in SP-141 was replaced with a fluorine atom, was also synthesized and tested for its MDM2 binding affinity.

**Scheme 2:**
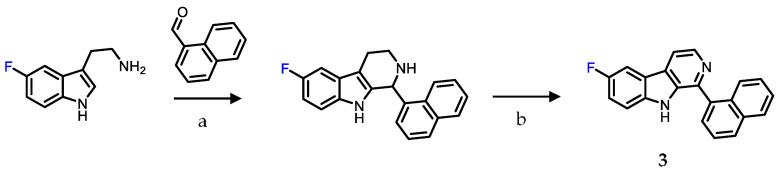
Synthesis scheme for the fluoro-β-carboline derivative **3**. Reaction conditions: (**a**) TFA, 0 °C → RT, THF, overnight; (**b**) Pd/C, xylenes, reflux, 4 h.

First, the parent compound SP-141 (**5**) was synthesized following a reported procedure and converted to the phenol precursor **6** by demethylation of the methoxy function using boron tribromide in dichloromethane [28]. Next, the fluoroethoxy and fluoropropoxy derivatives **1** and **2** were synthesized by alkylation of the phenol precursor **6** using fluoroethyl bromide and fluoropropyl tosylate, in 17% and 52% yields, respectively (Scheme 1). The fluoro-β-carboline derivative **3** was synthesized similar to the methoxy derivative **5**, but using 5-fluorotryptamine hydrochloride as the starting material in place of the 5-methoxytryptamine hydrochloride employed for **5**, with an overall yield of 26% (Scheme 2).

### 2.2. Surface Plasmon Resonance (SPR) Analysis

The ability of the synthesized nonradioactive fluorinated analogues **1**–**3** to bind to the MDM2 protein was assessed by SPR analysis using a commercially available recombinant human MDM2 protein (E3-204, Novus Biologicals [29]) and varying concentrations of the test compounds (1.25–20 µM) [24,30]. For these experiments, the MDM2 protein was immobilized at a high density (~12,900 Response Units) on a series S sensor chip CM5 (GE Healthcare), and samples were run in tris-buffered saline (TBS) with 2% DMSO as a co-solvent. An unmodified CM5 sensor surface was utilized as a reference. Two other known inhibitors of MDM2—RG7388 and a stapled peptide, VIP116—were also included in the assay as reference compounds, in addition to SP-141 [30,31]. 

**Figure 2:**
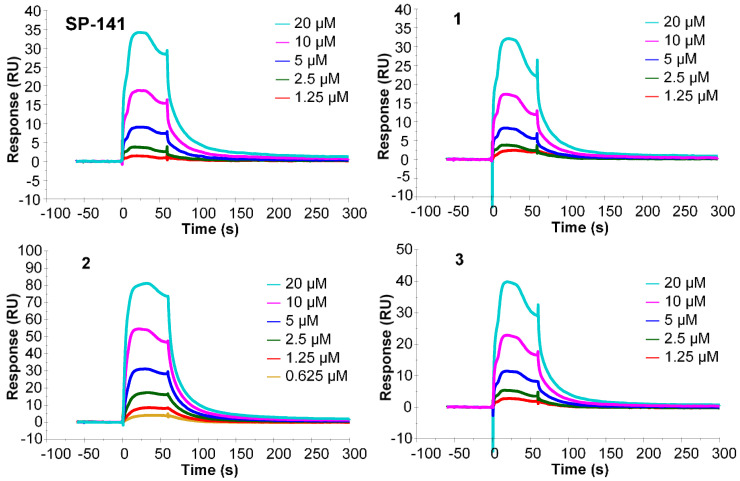
Surface plasmon resonance (SPR) analysis of the interaction of SP-141 and the newly synthesized fluorinated analogues **1**–**3** with human recombinant MDM2 protein [29]. Binding response curves recorded at 25 °C for varying concentrations of the test compounds (1.25–20 µM).

In initial screening SPR experiments, all of the compounds, including the reference MDM2 inhibitors, demonstrated fairly weak binding responses at nanomolar concentrations towards the MDM2 protein. Subsequent screening studies employing higher concentrations of the test compounds (2 µM and 10 µM) yielded binding responses within the anticipated range based on the surface density of the protein and the expected theoretical maximum binding responses. Therefore, subsequent titration analyses were done using serial two-fold dilutions of the test compounds at 1.25–20 µM. Binding curves were both surface- and buffer-reference subtracted. The results from the titration analyses revealed a concentration-dependent increase in binding response for all of the tested compounds (Figure 2 and Figure S1). Furthermore, the binding responses of the nonradioactive fluorinated analogues **1**–**3** were comparable to the parent molecule SP-141, suggesting that substitution of the methoxy group at the C-6 position of the β-carboline moiety with a longer fluoroalkoxy function or a fluorine did not compromise the binding of these molecules to the MDM2 protein.

### 2.3. Radiolabeling

**Scheme 3:**
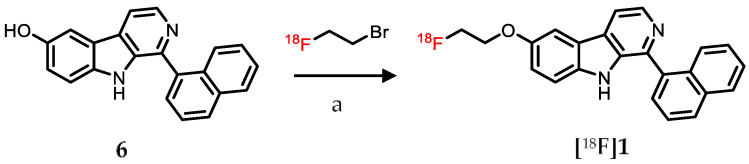
Fluorine-18 (^18^F) labeling of **1** using the phenol precursor **6** and [^18^F]fluoroethyl bromide as the labeling reagent. Reaction conditions: (**a**) DMF, sodium hydroxide (1N), 140 °C, 20 min.

To assess the suitability of the SP-141 molecular scaffold for radiolabeled MDM2 probe development, the fluoroethoxy derivate **1** was labeled with ^18^F using the readily available phenol precursor **6** (Scheme 1) and [^18^F]fluoroethyl bromide ([^18^F]FEtBr) as previously described [32]. Fluorine-18 labeling of **1** was achieved similar to its nonradioactive synthesis by reacting the phenol precursor **6** with [^18^F]FEtBr but using sodium hydroxide as the base instead of the cesium carbonate employed in the nonradioactive synthesis (Scheme 3). The labeled compound was purified by reversed-phase HPLC, and quality control was performed by analytical HPLC. [^18^F]**1** was obtained in an isolated (decay corrected) radiochemical yield of 24 ± 10% (*n* = 5) relative to the initial [^18^F]FEtBr activity, with an average molar activity of 17.5 GBq/µmole and a radiochemical purity > 98%. The identity of the labeled compound was confirmed by co-injection with the nonradioactive reference fluoro analogue **1** on the analytical HPLC system (*t*_R_ = 5.5 and 5.4 min, respectively; Figure S2). 

### 2.4. Lipophilicity and In Vitro Stability of *[^18^F]**1*** in Human Serum

The lipophilicity of [^18^F]**1** was determined by its partitioning between PBS (pH 7.4) and *n*-octanol by the shake-flask method as previously described [33]. In these experiments, the labeled compound displayed a log *D*_7.4_ of 2.35 ± 0.19, which should facilitate the passive permeation of [^18^F]**1** through cell membranes. 

**Figure 3:**
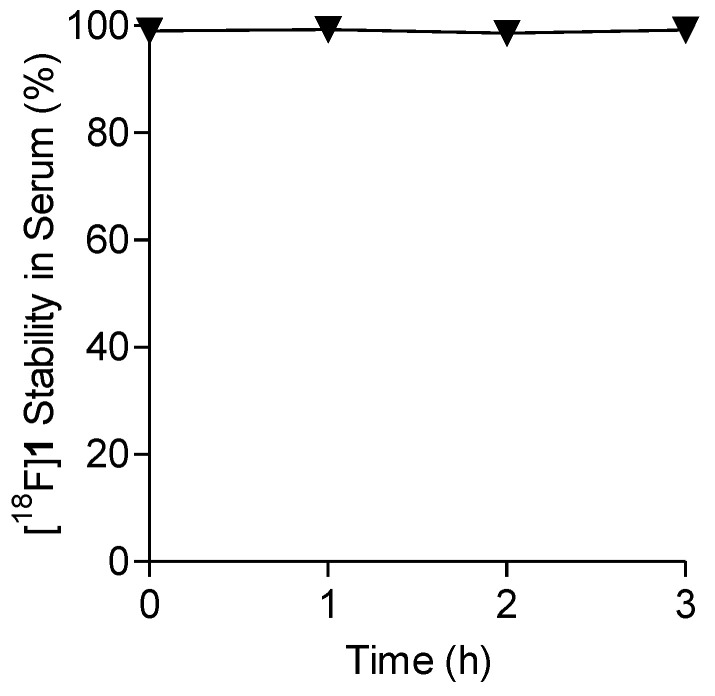
Percentage of intact [^18^F]**1** in human serum after 1–3 h incubation.

Next, the in vitro stability of [^18^F]**1** was examined by incubating the labeled compound with human serum for up to 3 h at 37 °C, followed by HPLC analysis of the samples using gradient elution conditions. For all of the tested time points (1, 2, and 3 h), the percentage of the intact [^18^F]**1** remained ~100%, indicating that the labeled compound is stable in human serum in vitro for up to 3 h (Figure 3 and Figure S3). 

### 2.5. Uptake in MDM2 Expressing Tumor Cell Lines

The ability of [^18^F]**1** to bind to MDM2 expressing human tumor cells was assessed using two wild-type p53 cell lines, MCF-7 breast adenocarcinoma, and HepG2 hepatocellular carcinoma. These cell lines have significant MDM2 expression levels and have been utilized previously for assessing potential MDM2 inhibitors, including SP-141 [12,24,25]. Additionally, previous studies have shown that treatment of MCF-7 and HepG2 cells with SP-141 at 0.5–1.0 µM concentration for 24 h decreases cellular MDM2 levels by inducing autoubiquitination and proteasomal degradation of the MDM2 protein independent of p53 mutational status [24,25]. Thus, to examine the uptake and specificity of [^18^F]**1** by MDM2 expressing tumor cell lines, MCF-7 and HepG2 cells were treated with 0, 1, 5, or 10 µM SP-141 for 21 h, and then assessed for [^18^F]**1** uptake for 1 h. Nutlin-3, a well-established small molecule inhibitor of MDM2, was used as a negative control because it does not induce MDM2 degradation and thus, should not decrease [^18^F]**1** uptake in the aforementioned tumor cell lines after an overnight treatment [14]. 

**Figure 4:**
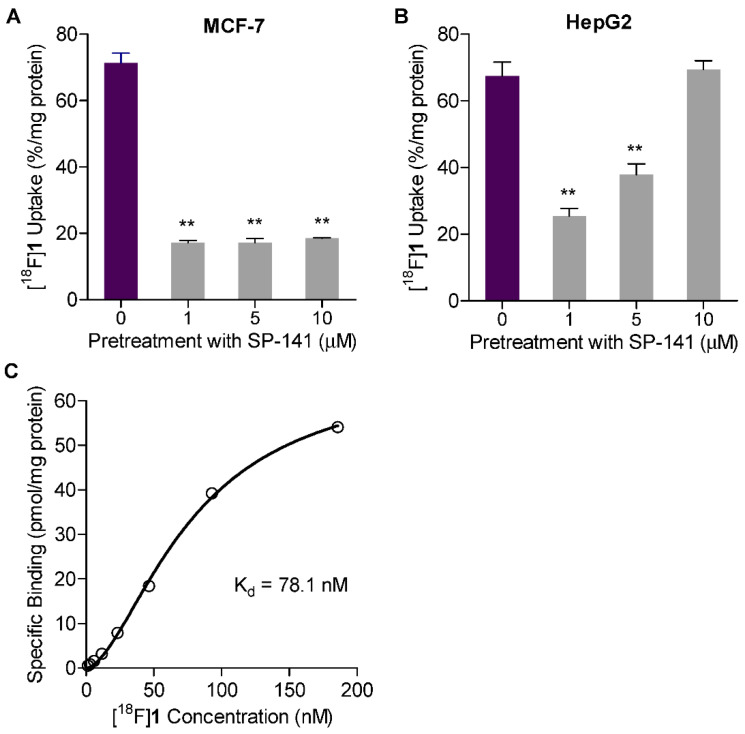
Uptake and the specificity of [^18^F]**1** in MDM2 expressing wild-type p53 tumor cell lines, (**A**) MCF-7 and (**B**) HepG2. Cells were pretreated with varying concentrations of SP-141 (1–10 µM) for 21 h to induce MDM2 degradation and then assessed for [^18^F]**1** uptake for 1 h. (**C**) Binding affinity curve for [^18^F]**1** on MCF-7 cells. MCF-7 cells were incubated with increasing concentrations of [^18^F]**1**, and the uptake was determined at 30 min post incubation. Nonspecific binding was assessed in parallel in cells that were exposed to SP-141 (1 µM) for 25 h to induce MDM2 degradation. Data are shown as mean ± SD for triplicates. ** *p* < 0.01 vs. no SP-141 pretreatment (0 µM).

As anticipated, a significant decrease in [^18^F]**1** uptake in SP-141-treated MCF-7 and HepG2 cells was observed compared to vehicle-treated cells (Figure 4A,B). The uptake of [^18^F]**1** in SP-141-treated MCF-7 cells was ≤18.5 ± 0.0 %/mg protein compared to 71.4 ± 0.0 %/mg protein in vehicle-treated control cells (*p* < 0.05). Similar results were observed in the HepG2 cell line both in terms of baseline levels of [^18^F]**1** uptake (~68 %/mg protein) and the decline in uptake with 1 or 5 µM SP-141 treatment. However, HepG2 cells exhibited a trend towards a decreasing effect of SP-141 as its concentration increased from 1 µM to 10 µM, with no noticeable effects of 10 µM SP-141 on [^18^F]**1** uptake (Figure 4B). In general, these results are consistent with those reported previously on the ability of SP-141 to decrease MDM2 levels in these cell lines and indicate that 1 µM is the optimal SP-141 concentration for inducing MDM2 degradation in vitro [24,25]. In contrast, Nutlin-3 treatment did not show any inhibitory effects on [^18^F]**1** uptake in either cell line. Furthermore, consistent with its mechanism of activity and its potential for activating p53-transcriptional genes, including MDM2 [14], Nutlin-3 pretreatment resulted in significant increases in [^18^F]**1** uptake in MCF-7 cells (at 1 and 5 µM Nutlin-3) and HepG2 cells (at 10 µM Nutlin-3; Figure S4), indicating that [^18^F]**1** uptake was MDM2-dependent.

### 2.6. Saturation Binding Assay

Next, saturation binding assays were conducted to evaluate the binding affinity of [^18^F]**1** on MCF-7 cells. In these experiments, MCF-7 cells seeded in 24-well plates were incubated for 30 min with eight increasing concentrations of [^18^F]**1** in serial two-fold dilutions (185.5–1.4 nM). Nonspecific binding was assessed in parallel using cells that were pretreated with 1 µM SP-141 for about 25 h to induce MDM2 degradation, thereby depleting MDM2 from the cells. Uptake of [^18^F]**1** in SP-141-pretreated cells was considered nonspecific and was subtracted from the total binding data for untreated cells at each radiotracer concentration to calculate the equilibrium dissociation constant (*K*_d_) by nonlinear regression analysis in Graphpad Prism^®^. Figure 4C shows the specific binding data for [^18^F]**1**, expressed in picomoles per milligram protein and plotted against [^18^F]**1** concentration (nM). Nonlinear regression analysis of the saturation binding data revealed a *K*_d_ of 78.1 nM, with a Hill slope of 1.69, for [^18^F]**1** on MCF-7 cells. 

### 2.7. Measuring Changes in *[^18^F]**1*** Uptake in Response to MDM2 Inhibition with SP-141

**Figure 5:**
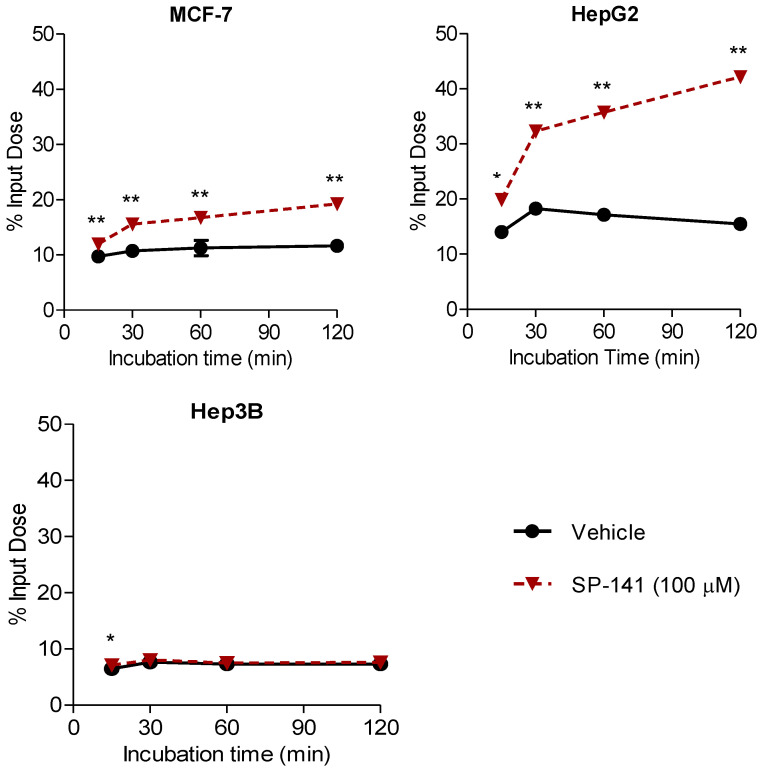
Changes in [^18^F]**1** uptake in tumor cells in response to MDM2 inhibition with SP-141 at 100 µM concentration. MCF-7, HepG2, and Hep3B cells were incubated with [^18^F]**1** with or without SP-141 (100 µM), and the uptake was assessed after 15–120 min. Co-incubation of cells with 100 µM SP-141 resulted in a significant increase in [^18^F]**1** uptake in wild-type p53 cell lines MCF-7 and HepG2 but not in the p53 null cell line Hep3B, potentially reflecting changes in MDM2 levels as a result of MDM2:p53 inhibition by SP-141. ** *p* < 0.01 and * *p* < 0.05 vs. the corresponding vehicle-treated cells.

Because MDM2 is a transcriptional target of p53, stabilization of p53 levels after MDM2 inhibition with SP-141 is expected to cause a temporary increase in MDM2 protein expression via the autoregulatory loop [34]. Thus, we hypothesized that treatment of tumor cells with high concentrations of SP-141 for shorter periods (e.g., <4 h) might induce MDM2 expression as a consequence of p53 activation, whereas low concentrations of SP-141 (≤1 µM) and longer incubation times (≥20 h) appear to promote MDM2 degradation in vitro. To investigate this, MCF-7 and HepG2 cells were incubated with or without 100 µM SP-141 for 15–120 min, and uptake was measured as a percentage input dose for each time point. Hep3B, a p53 null cell line, was used as a negative control. The results from these experiments showed a continuous increase in [^18^F]**1** uptake in the presence of 100 µM SP-141 (Figure 5). At 2 h post incubation, the uptake of [^18^F]**1** in MCF-7 cells and HepG2 cells increased by about 1.7 and 2.7-fold, respectively, compared to vehicle only cells. In contrast, the addition of SP-141 had no effect on the uptake of [^18^F]**1** on negative-control Hep3B cells. The increased [^18^F]**1** uptake in the wild-type p53 cell lines MCF-7 and HepG2 likely reflect MDM2 induction resultant from p53 activation by SP-141, whereas the Hep3B (p53^-/-^) cell line was unable to do so because this cell line lacks a functional p53 protein. 

### 2.8. Tissue Distribution Studies in HepG2 Xenografts

Based on the promising results obtained with [^18^F]**1** in vitro, tissue distribution studies were conducted in athymic mice bearing subcutaneous HepG2 tumor xenografts. HepG2 tumor model was selected because of the higher uptake of [^18^F]**1** in HepG2 cells and a substantial increase in the uptake in response to co-incubation with 100 µM SP-141, compared to that in MCF-7 cells (Figure 5) [25,35]. Groups of five mice were injected intravenously with [^18^F]**1** and euthanized at 0.5, 1, or 2 h post injection to determine the tissue distribution of [^18^F]**1**. A separate group of animals (*n* = 4) were injected intraperitoneally with SP-141 (1.2 mg per mouse; ~140 µM) 30 min before [^18^F]**1** injection to assess the specificity of tracer uptake in tumors. As shown in Table 1, [^18^F]**1** had slow clearance from the blood pool, decreasing only slightly from 4.2 ± 0.6 %ID/g at 30 min to 3.3 ± 0.8 %ID/g at 2 h post injection. At 30 min post injection, radioactivity levels in most organs were ≥3 %ID/g and decreased to ~2 %ID/g by 2 h. At 2 h post injection, ^18^F activity was highest in the large intestine at 7.1 ± 2.9 %ID/g, suggesting a hepatobiliary route for [^18^F]**1** clearance, albeit at a slow rate. Uptake of ^18^F activity in the bone increased from 3.1 ± 0.6 %ID/g at 30 min to 5.4 %ID/g at 2 h, indicating that some defluorination of the labeled compound occurred in vivo.

With respect to tumor uptake, the concentration of [^18^F]**1** in HepG2 xenografts was 3.3 ± 0.3 %ID/g at 30 min and remained essentially unchanged during the 2-h observation period. Treatment of mice with SP-141 (1.2 mg/mouse) 30 min before [^18^F]**1** injection did not significantly change [^18^F]**1** tumor uptake at 1 h post injection compared with the untreated group. However, SP-141-treated mice exhibited significantly lower levels of radioactivity in blood and bone compared to the untreated group, suggesting that unlabeled SP-141 altered blood clearance of [^18^F]**1** and potentially its metabolism in vivo. Contrary to our expectations, tumor-to-normal tissue ratios for [^18^F]**1** remained <2:1 at 2 h post injection, including in muscle (1.14 ± 0.18). Thus, the labeled compound does not appear to have optimal characteristics for in vivo use in its current form.

## 3. Discussion

In this work, we investigated the suitability of SP-141 as a molecular scaffold for developing radiolabeled agents for imaging MDM2 expression in tumors by PET. Our attempts to determine the binding affinity of the MDM2 inhibitors, including reference compounds by SPR analysis using a commercially-available protein preparation, yielded detectable binding responses only in the micromolar concentration range (1.25–20 µM; Figure 2 and Figure S1). This was unexpected given the nanomolar affinity range for the reference compounds SP-141, RG7388, and VIP116 [24,30,31]. The specific binding affinities previously reported for the aforementioned reference compounds [24,30,31] were not replicated in this SPR study. We suspect that this may be due to the fact that the protein preparation (full length MDM2) that we used was not ideally suited for SPR, as only a subset of the molecules in the product were active as assessed by autoubiquitination assays (personal communication with Bio-Techne Corporation). Using a different protein format (e.g., MDM2 protein fragment [30,31]), conjugation chemistry and/or sensor chip to capture higher levels of MDM2 protein may enhance the sensitivity of the SPR assay to allow recording of binding responses in the nanomolar range for the test compounds [30]. Nonetheless, all of the new fluoro analogues (**1**–**3**) showed concentration-dependent increases in reference-subtracted binding response toward the MDM2 protein, and importantly, their responses were comparable to those observed for the parent molecule SP-141 (Figure 2). Combined with the use of RG7388 and VIP116 as internal reference compounds (Figure S1), this confirmed the ability of the nonradioactive fluoro analogues **1**–**3** to bind to MDM2 protein. 

Previous studies identified SP-141 as a potent MDM2 inhibitor (*K*_i_ = 28 nM) based on SAR studies and its anti-tumor activity in multiple human tumor xenografts, including MCF-7 and HepG2 [24,25,28]. In MCF-7 and HepG2 xenograft models, intraperitoneal administration of SP-141 at a 40 mg/kg dose for 5 days a week decreased MDM2 levels in tumors and inhibited tumor growth significantly after 42 and 20 days of treatment, respectively. While most small molecule MDM2 inhibitors are reported to exert therapeutic activity by blocking the interaction of MDM2 with p53, SP-141 appears to possess a unique and incompletely understood mode of action against MDM2. Studies suggest that SP-141 may bind to the p53 binding pocket of the MDM2 but can also induce MDM2 degradation, an activity that is not observed with other small molecule MDM2 inhibitors, such as Nutlin-3 or RG7388 [14,24,25]. Consistent with literature reports, treatment of MCF-7 cells with 1 µM SP-141 for 21 h in the present study decreased uptake of [^18^F]**1** by 76% compared to vehicle-treated cells (Figure 4A). Based on these data, we selected 1 µM as the concentration of SP-141 for the saturation binding assays and for depleting MDM2 in MCF-7 cells to measure nonspecific binding for [^18^F]**1**. These experiments yielded a binding affinity of 78.1 nM for the labeled compound. Differences in the type of assay, protein source, and other variables, such as the protein capture method, preclude direct comparison of our cell-based binding affinity of [^18^F]**1** with those reported in the literature for the reference MDM2 inhibitors employed in this study. We note that the binding affinity of SP-141 and RG7388 determined by SPR analysis were reported to be 43 nM and 9.79 nM, respectively [24,30]. Similarly, the binding affinity of the stapled peptide VIP116, determined in competition with a p53-based peptide in fluorescence anisotropy experiments, was reported to be 15.1 nM [31]. Compared with these MDM2 inhibitors, [^18^F]**1** appeared to have a somewhat lower binding affinity for the MDM2 protein. It seems likely that improvements in binding affinity will be needed to achieve high uptake and retention in MDM2 expressing tumors in vivo.

As noted above, MDM2 itself is a transcriptional target of p53, and thus, in addition to suppressing tumor growth, p53 activation is expected to cause a temporary increase in MDM2 expression levels in tumor cells [34]. Hence, MDM2 and other p53-regulated genes (e.g., p21) are frequently used as pharmacodynamic biomarkers for assessing treatment response to MDM2 inhibitors [13,16]. Thus, we examined the ability of [^18^F]**1** to track changes in MDM2 expression levels by co-incubating MCF-7 or HepG2 cells with [^18^F]**1** and a pharmacological concentration of SP-141 (100 µM) in vitro for shorter time periods (up to 2 h). These treatment conditions are in contrast to those needed for inducing MDM2 degradation in vitro with SP-141, namely low drug concentration (1 µM SP-141) and a longer incubation period (>20 h) [24,25]. In the presence of 100 µM SP-141, the uptake of [^18^F]**1** increased with time, by 1.2-fold at 15 min and 1.8-fold at 2 h for MCF-7 cells and by 1.4-fold at 15 min and 2.9-fold at 2 h for HepG2 cells, compared to cells incubated with only [^18^F]**1** (Figure 5). With regard to the time needed for MDM2 induction post stabilization of p53, it has been reported that MDM2 protein levels can peak 1.5–2 h after exposure of tumor cells to ionizing radiation, which causes DNA damage and accumulation of p53 protein within 1 h after irradiation [4]. With respect to the magnitude of increase in MDM2 levels after treatment with MDM2 inhibitors, AML patients treated with RG7112 exhibited a 2.8-fold increase in median MDM2 gene expression levels in peripheral blasts after 10 days of treatment. Although our results need to be corroborated by measuring MDM2 expression levels by Western blotting or other appropriate methods, they are consistent with the literature and the expected p53-mediated induction of MDM2 after stabilization of p53 by MDM2 inhibitors in wild-type p53 cell lines (MCF-7 and HepG2) but not in p53 mutant or null cell lines (Hep3B). 

The cell uptake and saturation binding data determined in this study confirmed the specificity of [^18^F]**1** uptake in the MDM2-expressing wild-type p53 MCF-7 and HepG2 cell lines. However, notwithstanding the promising data obtained with [^18^F]**1** in vitro, the labeled compound exhibited poor pharmacokinetics in mice bearing HepG2 tumor xenografts. At 30 min post injection, [^18^F]**1** exhibited rather uniform distribution among different tissues, with about 3–4 %ID/g in most of the organs and in tumors. Although the tumor uptake remained nearly stable (~3 %ID/g), and the radioactivity levels in the normal tissues decreased slightly (to 2–3 %ID/g), the tumor-to-background ratios at 2 h post injection were fairly low (<2.0). While the slow pharmacokinetics exhibited by [^18^F]**1** were unexpected based on its near-optimal lipophilicity (log *D*_7.4_ = 2.35), the poor clearance of ^18^F activity from normal tissues could be due to other factors, such as nonspecific retention or formation of metabolites that have increased residence times in normal tissues [36]. Additionally, pretreatment of animals with an excess of SP-141 at ~140 µM (1.2 mg/mouse) in the biodistribution study did not yield any blocking effects nor increase in the uptake of [^18^F]**1** due to p53 activation (3.2 ± 0.7 %ID/g vs. 3.0 ± 0.7 %ID/g for the untreated group; Table 1). While these results have precluded establishing the specificity of [^18^F]**1** uptake in tumors and further evaluation of the labeled compound by PET imaging in vivo, it is possible that the 30 min pretreatment time we used for SP-141 was not sufficient to induce MDM2 levels via p53 activation. Thus, similar to the in vitro setting where the maximum increase in [^18^F]**1** uptake with 100 µM SP-141 was observed at 2 h post incubation, a longer pretreatment schedule (e.g., ≥2 h) may be beneficial in vivo as well. Taken together, the promising in vitro data obtained with [^18^F]**1** in the present study supports radiolabeling and evaluation of the two other fluorinated analogues, **2** and **3**, synthesized in this work. It is also worth considering other potent MDM2 inhibitor scaffolds for radiotracer development. To that end, efforts are currently underway to synthesize and evaluate radiolabeled derivatives of clinically validated MDM2 inhibitors (e.g., RG7388), the results of which will be reported in subsequent publications.

## 4. Materials and Methods

All chemicals, reagents, and solvents used in this work were obtained from commercial vendors and used as supplied. Recombinant human MDM2 protein was purchased from Novus Biologicals, Bio-Techne Brands (Centennial, CO, USA; Cat. #E3-204-050; 0.83 mg/mL) and used as provided. The stapled peptide VIP116 was kindly provided by Dr. Fernando Ferrer from p53Lab at the Agency for Science, Technology, and Research (A*STAR), Singapore. RG7388 was purchased from Ontario Chemicals, Inc. (Ontario, Canada). Purification of all intermediates and final compounds in the chemical syntheses was achieved using a Biotage Isolera™ flash purification system (Biotage, Charlotte, NC, USA) and SNAP Ultra silica cartridges (10 g or 25 g, Biotage, Charlotte, NC, USA). Semi-preparative purification of [^18^F]**1** was achieved using the Beckman Gold HPLC system that consisted of a gradient solvent delivery module, variable wavelength UV detector set at 254 nm, and a radiometric detector (Bioscan Analytical Instruments). Data were acquired and analyzed using 32 Karat^®^ software (Beckman). Quality control analysis of [^18^F]**1** was performed on an analytical HPLC system that consisted of an isocratic HPLC pump (Knauer, Germany) connected with an XBridge C_18_ column (3.5 μm, 3.0 × 100 mm; Waters). The effluent was passed through a UV/VIS detector set at 254 nm (Knauer) and a radiometric detector (Carroll & Ramsey Associates, Berkeley, CA, USA). Chromatograms were recorded and analyzed using PeakSimple software (SRI Instruments, Torrance, CA, USA). NMR spectra were recorded on a Varian 500 MHz or 400 MHz spectrometer (Palo Alto, CA, USA). Mass spectrometry (HRMS) analysis was performed using an Agilent LC/MS-TOF (ESI). MCF-7 cells were cultured in Minimum Essential Medium (MEM, Gibco™, Gaithersburg, MD, USA) supplemented with human recombinant insulin (0.01 mg/mL), fetal bovine serum (10%), and penicillin-streptomycin (1%). The hepatocellular carcinoma cell lines HepG2 and Hep3B were cultured in Dulbecco’s modified Eagle medium (DMEM, Gibco) supplemented with fetal bovine serum (10%). All cell lines were cultured and maintained under standard cell culture conditions (5% CO_2_, 37 °C).

### 4.1. Chemistry

6-Methoxy-1-(naphthalen-1-yl)-2,3,4,9-tetrahydro-1H-pyrido[3,4-b]indole (**4**). 1-napthaldehyde (0.68 mL, 6 mmol, 1.2 eq.) was added to a stirred solution of 5-methoxytryptamine hydrochloride (1.13 g, 5 mmol) in tetrahydrofuran (100 mL). Subsequently, the reaction mixture was cooled to 0–4 °C in an ice bath, and trifluoroacetic acid (TFA, 1.9 mL) was added in a dropwise manner. After 2–3 h of stirring in an ice bath, the reaction mixture was allowed to warm to room temperature and stirring was continued overnight. The reaction was quenched with sodium bicarbonate (50 mL), and the crude mixture was concentrated under reduced pressure, followed by extraction with ethyl acetate (2 × 50 mL). The organic layers were combined, rinsed with water, dried over sodium sulfite, and concentrated under reduced pressure to obtain the tetrahydro-β-carboline derivative **4**. The residue was dried under vacuum for 6 h and used for the next step without further purification.

6-Methoxy-1-(naphthalen-1-yl)-9H-pyrido[3,4-b]indole (**5**, SP-141). Palladium on carbon (Pd/C, 0.5 g) was added to the above dried residue dissolved in xylenes (100 mL), and the resulting mixture was heated under reflux conditions for 4 h. The crude mixture was filtered, rinsed with ethyl acetate, and the filtrate was concentrated under reduced pressure. Next, the residue was dissolved in ethyl acetate (50 mL) and rinsed with water (2 × 50 mL). The organic layer was dried over sodium sulfite and concentrated under reduced pressure. The crude product was purified by flash column chromatography using ethyl acetate and hexanes (0→70%) to obtain compound **5** in 33% yield (0.54 g). ^1^H-NMR (500 MHz, CDCl_3_): *δ* 8.61 (d, *J* = 5.5 Hz, 1H), 8.00–7.91 (m, 4H), 7.76–7.71 (m, 2H), 7.64–7.61 (m, 2H), 7.52–7.38 (m, 2H), 7.26–7.14 (m, 2H), 3.93 (s, 3H). HRMS (ESI) calculated for C_22_H_16_N_2_O [M + H]^+^), 325.1335; found, 325.1339.

1-(Naphthalen-1-yl)-9H-pyrido[3,4-b]indol-6-ol (**6**). Boron tribromide in dichloromethane (1 M, 4.95 mL) was added in a dropwise manner to a stirred solution of compound **5** (0.52 g, 1.6 mmol) in dichloromethane (15 mL) at −70 °C. The reaction mixture was allowed to warm to room temperature and stirred for 3 days, after which time thin-layer chromatography (TLC) analysis showed complete conversion of the starting material to a more hydrophilic compound. Next, solvents were evaporated under reduced pressure, and the crude product was rinsed with methanol, and the residue was purified by flash column chromatography using dichloromethane and methanol (0→8%) to obtain the phenol precursor **6** in 82% yield (0.41 g). ^1^H-NMR (500 MHz, DMSO-d_6_): δ 10.02 (bs, 1H), 9.60 (1H), 8.77–8.59 (m, 2H), 8.33 (d, *J* = 7.5 Hz, 1H), 8.19 (d, *J* = 7.5 Hz, 1H), 7.97 (d, *J* = 6.0 Hz, 1H), 7.84–7.81 (m, 2H), 7.67 (m, 1H) 7.55–7.44 (m, 3H) 7.28 (d, *J* = 7.5 Hz, 1H). HRMS (ESI) calculated for C_21_H_14_N_2_O [M + H]^+^, 311.1184; found, 311.1185.

6-(2-Fluoroethoxy)-1-(naphthalen-1-yl)-9H-pyrido[3,4-b]indole (**1**). Cesium carbonate (115 mg, 0.35 mmol) and 2-fluoroethyl bromide (63 µL, 0.48 mmol) were added to a stirred solution of the phenol derivative **6** (100 mg, 0.32 mmol) in dimethylformamide (10 mL). The reaction mixture was stirred at 70 °C for 24 h after which time HPLC analysis of the reaction mixture showed ~35% conversion to a more lipophilic compound. Next, solvents were evaporated and the resulting crude product was purified by flash column chromatography using ethyl acetate and hexanes (0→80%) to obtain the fluoroethoxy analogue **1** in 17% yield (20 mg). ^1^H-NMR (400 MHz, CDCl_3_): *δ* 8.62 (d, *J* = 5.2, 1H), 8.03–7.98 (m, 3H), 7.80 (d, *J* = 8.0, 1H), 7.71 (d, *J* = 8.8, 1H), 7.67–7.63 (m, 2H), 7.55 (m, 1H), 7.43 (m, 1H), 7.24–7.32 (m, 2H), 4.79 (m, *J* = 47.4, 2H), 4.37 (m, *J* = 28.4, 2H). ^13^C-NMR (125 MHz, CDCl_3_): *δ* 153.4, 136.0, 135.5, 134.3, 131.3, 129.8, 128.9, 128.1, 127.1, 126.5, 125.9, 125.6, 125.9, 125.6, 122.2, 119.8, 114.2, 112.8, 105.5, 83.0, 81.6, 68.7, 68.5. HRMS (ESI) calculated for C_23_H_17_FN_2_O [M + H]^+^, 357.1398; found, 357.1406.

6-(3-Fluoropropoxy)-1-(naphthalen-1-yl)-9H-pyrido[3,4-b]indole (**2**). Cesium carbonate (0.24 mmol, 78.75 mg) and 3-fluoropropyl-4-methylbenzenesulfonate (56.2 mg, 0.24 mmol) were added to a stirred solution of the phenol precursor **6** (50 mg, 0.16 mmol) in methanol (3 mL). The resulting mixture was heated at 70 °C for 19 h, after which time TLC analysis in ethyl acetate and hexanes (50:50) revealed a lipophilic spot. Solvents were evaporated and the resulting residue was adsorbed onto silica gel and purified by flash column chromatography using ethyl acetate and hexanes (0→100%) to obtain the fluoropropoxy derivative **2** in 52% yield (31 mg). ^1^H-NMR (400 MHz, CDCl_3_): *δ* 8.60 (d, *J* = 5.2, 1H), 8.02–7.95 (m, 4H), 7.79 (d, *J* = 7.2, 1H), 7.71 (d, *J* = 9.6, 1H), 7.64 (m, 2H), 7.53 (m, 1H), 7.42 (m, 1H), 7.29–7.17 (m, 2H), 4.71 (m, *J* = 47.6, 2H), 4.235 (m, 2H), 2.20–2.29 (m, 2H). ^13^C-NMR (125 MHz, CDCl_3_): *δ* 153.4, 135.3, 134.01, 131.1, 129.4, 128.6, 127.6, 126.7, 126.2, 125.6, 125.5, 122.1, 119.1, 113.9, 112.4, 104.9, 81.5, 80.2, 64.6, 64.5, 30.65, 30.5. HRMS (ESI) calculated for C_24_H_19_FN_2_O [M + H]^+^, 371.1554; found, 371.1563.

6-Fluoro-1-(naphthalen-1-yl)-9H-pyrido[3,4-b]indole (**3**). The fluoro-β-carboline derivative **3** was synthesized via a two-step scheme similar to the methoxy derivative **5** (SP-141), but using 5-fluorotryptamine hydrochloride (0.5 g, 2.33 mmol) as the starting material. The crude product was purified by flash column chromatography using ethyl acetate and hexanes (0→50%) to obtain compound **3** as a brown solid (0.19 g, 26%). ^1^H-NMR (400 MHz, CDCl_3_): δ 8.62 (d, *J* = 5.6, 1H), 8.18 (bs, 1H), 8.02–7.97 (m, 3H), 7.85 (d, *J* = 8.4, 1H), 7.78 (d, *J* = 6.8, 1H), 7.71 (d, *J* = 8.8, 1H), 7.64 (m, 1H), 7.54 (m, 1H), 7.43 (m, 1H), 7.31–7.28 (m, 2H). ^13^C-NMR (125 MHz, CDCl_3_): *δ* 158.8, 156.9, 143.3, 139.1, 136.8, 135.9, 134.3, 131.3, 129.7, 128.8, 127.9, 127.0, 126.5, 125.8, 125.6, 122.4, 117.2, 114.3, 112.6, 107.5, 107.3. HRMS (ESI), calculated for C_21_H_13_FN_2_ [M + H]^+^, 313.1136; found, 313.1144.

### 4.2. Surface Plasmon Resonance (SPR) Analysis

The binding interaction between the synthesized nonradioactive fluorinated analogues and MDM2 protein was assessed by SPR on a Biacore™ T200 system (G.E. Healthcare) at 25 °C in TBS (tris-buffered saline) running buffer with 2% DMSO. For these assays, recombinant human MDM2 protein (catalog #E3-204-50, Novus Biologicals, Centennial, CO, USA) was covalently immobilized on a Series S Sensor Chip CM5 (G.E. Healthcare, Marlborough, MA, USA) via an amine coupling reaction following standard procedures recommended by the vendor. The MDM2 protein solution (0.8 mg/mL) was injected over the surface of the chip to achieve the desired immobilization response (~12,900 RU). Next, binding titration analysis was performed by sequential 60-s injections of different concentrations (0.625–20 µM, serial two-fold dilutions) of the test compounds, followed by a 420-s dissociation phase, at 50 µL/min. Surfaces were regenerated between analyte injections with a 30-s injection of 10 mM glycine-HCl, pH 2.0, at a flow rate of 50 µL/min. Binding profile curves with reference subtracted were obtained using an unmodified CM5 surface as a reference surface and running buffer (TBS, 2% DMSO) as a reference sample. Data analysis was performed using Biacore™ T200 Evaluation Software (G.E. Healthcare).

### 4.3. Radiochemistry

Fluorine-18, trapped on a Sep-Pak^®^ Light Accell plus QMA cartridge (Waters Corporation, Milford, MA, USA), was eluted with a potassium carbonate (0.8 mg) and Kryptofix (8 mg) solution in methanol and water (9:1, 0.8 mL). Solvents were evaporated, and the residual water was removed by azeotropic distillation with acetonitrile (3 × 0.5 mL). The dried ^18^F activity was partially cooled, and 2-bromoethyl trifluoromethanesulfonate (5 µL, Enamine Ltd., Monmouth Jct., NJ, USA) dissolved in 1,2-dichlorobenzene (0.7 mL) was added to the vial. Then, the reaction vial was heated at 120 °C, and the resulting ^18^F-fluoroethyl bromide ([^18^F]FEtBr) was passed through an in-line C_18_ Sep-Pak^®^ cartridge to remove any traces of the solvent, using argon as a carrier gas. The distilled [^18^F]FEtBr activity was captured in a second reaction vessel containing the phenol precursor **6** (0.5 mg) dissolved in DMF (0.25 mL). After distilling sufficient [^18^F]FEtBr (15–20 min), sodium hydroxide (1 N, 5 µL) was added, and the reaction mixture heated at 140 °C for 15–20 min. The reaction mixture was cooled, diluted with water (0.75 mL), and purified by RP-HPLC using the Beckman Gold HPLC system consisting of an XBridge™ C18 column (4.6 × 150 mm) eluted with 40% ethanol in sodium acetate buffer (0.05 M, pH 5.5) at a flow rate of 1 mL per minute. The peak corresponding to [^18^F]**1** was collected (t_R_ = 25 min) and tested for radiochemical purity on the analytical HPLC system (Knauer) with acetonitrile and water (45:55; 0.1% TFA) as the eluant at a flow rate of 1 mL per minute (t_R_ = 5.5 min).

### 4.4. Determination of Lipophilicity of *[^18^F]**1***

An aliquot of [^18^F]**1** (0.37 MBq) was added to a mixture of PBS (pH 7.4) and *n*-octanol (2 mL each, *n* = 3) in polypropylene tubes. The mixture was vortexed for one minute, and the organic phase was separated by centrifugation (3000 rpm, 5 min). Next, aliquots of octanol (50 µL) and PBS (500 µL) were collected from each tube into preweighed Eppendorf^®^ tubes and measured for radioactivity in an automated gamma counter (Wallac Wizard™, Perkin Elmer, Waltham, MA, USA). Samples were weighed, and the weights were converted to volume according to solvent density. The partition coefficient value was calculated as the ratio of radioactivity (counts per minute (CPM)/mL) in octanol to that in the aqueous phase.

### 4.5. Cell Uptake Studies with *[^18^F]**1***

Cell uptake studies of [^18^F]**1** were conducted using MCF-7 and HepG2 cell lines. Briefly, cells plated in 24-well plates (200,000/well) were treated with varying concentrations of SP-141 (1, 5, or 10 µM) or vehicle for 21 h and incubated with a fresh medium containing [^18^F]**1** (~18.5 kBq in 0.5 mL medium) for 1 h. Next, the incubation medium was removed, cells were rinsed with cold PBS (3 × 0.5 mL) and lysed using Cell Culture Lysis Reagent (0.25 mL, Promega Corporation, Madison, WI, USA) followed by a rinse with PBS (0.125 mL/well). For each well, the supernatant, PBS washes, and the cell lysate were measured for radioactivity in an automated gamma counter (Wallac Wizard™, Perkin Elmer, Waltham, MA, USA). Subsequently, cell lysates were analyzed for protein concentration using the Bradford assay (Bio-Rad, Hercules, CA, USA). Cell uptake for each well was normalized to its protein content and expressed as % uptake/mg protein. Results are presented as mean ± SD for three wells.

### 4.6. Saturation Binding Assay with *[^18^F]**1***

For the saturation binding assay with [^18^F]**1**, MCF-7 cells plated in 24-well plates (200,000 cells/well) were treated for 25 h with vehicle (DMSO, <5%; plate #1, for total binding) or SP-141 (1 µM; plate #2, to determine nonspecific binding). Afterward, supernatants were removed, cells rinsed with PBS (0.5 mL) and incubated for 30 min with eight increasing concentrations of [^18^F]**1** (1.4–185.5 nM, *n* = 3 per concentration), prepared by serial two-fold dilutions of HPLC-purified [^18^F]**1** in fresh medium. Next, the supernatants were removed, and the cells were processed as described above for the cell uptake studies. At each concentration of [^18^F]**1**, nonspecific binding was determined in parallel (plate #2) in cells treated with 1 µM SP-141 to induce MDM2 degradation. Specific binding data were calculated by subtracting nonspecific binding from total binding and fit using the equation “Specific binding with Hill slope” in GraphPad Prism^®^ (GraphPad, v5.01). Results were plotted as picomoles [^18^F]**1** bound per mg protein vs. [^18^F]**1** concentration in nM.

### 4.7. Measuring Changes in *[^18^F]**1*** Uptake in Response to MDM2 Inhibition with SP-141 In Vitro

For these studies, MCF-7 (WT-p53), HepG2 (WT-p53), and Hep3B (p53 null) cells were plated in 24-well plates (20,000/well) and incubated with [^18^F]**1** (~18.5 kBq in 0.5 mL) with or without 100 µM SP-141 for 15–120 min under standard cell culture conditions. After the incubation, supernatants were removed, cells were washed with cold PBS (3 × 0.5 mL) and lysed using Cell Culture Lysis Reagent (0.25 mL, Promega, Madison, WI, USA), followed by a rinse with PBS (0.125 mL/well). The radioactivity in supernatants, wash fractions, and cell lysates was counted for each well using the gamma counter (Wallac Wizard). Cell uptake was calculated as the percentage of the input dose for the two treatment conditions (with/without 100 µM SP-141).

### 4.8. Serum Stability Analysis

The stability of [^18^F]**1** in human serum (Innovative Research Inc., Novi, MI, USA) was analyzed by HPLC using a Chromolith^®^ Performance RP-18e column (4.6 × 100 mm, Millipore Sigma, Burlington, MA, USA), eluted with gradient mixtures of sodium acetate buffer (0.05 M, pH 5.5) and ethanol. The percentage of ethanol was kept at 5% for the first 5 min at a flow rate of 0.5 mL and then increased to 90% over 15 min at a flow rate of 1 mL/min. The stability studies were initiated by incubating human serum (0.5 mL) with [^18^F]**1** (6.66 MBq in 20 µL ethanol) at 37 °C. Samples (100 µL) were analyzed by HPLC after 0, 1, 2, and 3 h incubation. At each time point, the serum sample was spiked with the nonradioactive **1** (10 µg) before HPLC analysis for identity confirmation of the parent peak ([^18^F]**1**).

### 4.9. Tissue Distribution Studies

All animal experiments were conducted in accordance with the regulations of the Duke University Institutional Animal Care and Use Committee (IACUC) under an IACUC-approved protocol. Tissue distribution studies were conducted in mice bearing subcutaneous HepG2 tumor xenografts. To generate the tumor model, male athymic mice (Jackson Labs, Farmington, CT, USA) were injected with 5 × 10^6^ HepG2 cells suspended in DMEM and Matrigel (Corning^®^, Corning, NY, USA, 1:1, 100 µL) in the flank; tissue distribution studies were initiated when the tumor volume reached 200 mm^3^. For these studies, groups of five mice were injected with [^18^F]**1** by the intravenous route and euthanized at 0.5, 1, or 2 h post injection (*n* = 5 per time point) to assess the tissue distribution of [^18^F]**1** as described previously [25]. A separate group of animals (*n* = 4) was injected with SP-141 (1.2 mg per mouse; ~140 µM) by intraperitoneal injection 30 min before [^18^F]**1** injection to assess the specificity of tracer uptake. The concentration of ^18^F activity was calculated as the percentage injected dose per gram tissue (%ID/g).

## 5. Conclusions

We have synthesized three fluorinated analogues of the preclinically validated MDM2 inhibitor SP-141 and labeled the fluoroethoxy derivative **1** with ^18^F using [^18^F]FEtBr. The labeled compound ([^18^F]**1**) showed high uptake and specific binding in MDM2 expressing wild-type p53 tumor cell lines, MCF-7 and HepG2. The uptake of [^18^F]**1** in these cells could be modulated by co-incubation with a high concentration of SP-141, potentially reflecting underlying changes in MDM2 expression levels resultant from p53 activation by SP-141. However, tissue distribution studies indicated suboptimal pharmacokinetics and low tumor-to-background ratios for [^18^F]**1** in mice bearing HepG2 tumors. The other two fluorinated analogues **2** and **3** showed MDM2 binding responses that are similar to or better than **1** in SPR analyses, warranting ^18^F-labeling and evaluation of **2** and **3** to identify a labeled compound with more favorable pharmacokinetics and uptake in MDM2 expressing tumors in vivo.

## Figures and Tables

**Table 1:** Biodistribution data for [^18^F]**1** in athymic mice bearing HepG2 xenografts in the flank area ^1^.

Organ	30 min	1 h	1 h Block ^2^	2 h
Lungs	3.9 ± 0.6	3.8 ± 0.7	3.5 ± 0.8	2.1 ± 0.8
Heart	4.1 ± 0.7	4.0 ± 0.7	3.7 ± 0.3	3.3 ± 0.9
Kidneys	3.7 ± 0.8	3.3 ± 0.6	3.8 ± 0.4	2.4 ± 0.6
Liver	3.2 ± 0.5	2.8 ± 0.4	3.3 ± 0.6	2.4 ± 0.5
Spleen	3.3 ± 0.4	2.7 ± 0.7	3.0 ± 0.6	2.1 ± 0.4
Stomach	5.1 ± 2.0	2.7 ± 0.8	1.6 ± 1.2	2.1 ± 0.7
Small Intestine	3.9 ± 1.0	4.0 ± 0.7	4.1 ± 0.3	3.0 ± 0.5
Large Intestine	6.1 ± 1.7	7.7 ± 1.8	3.4 ± 1.4 *	7.1 ± 2.9
Skin	3.7 ± 0.7	3.4 ± 0.5	3.0 ± 0.7	3.1 ± 0.4
Muscle	3.5 ± 0.8	2.6 ± 0.6	3.3 ± 0.4	2.6 ± 0.7
Blood	4.2 ± 0.6	4.1 ± 0.6	2.7 ± 0.9 *	3.3 ± 0.8
Bone	3.1 ± 0.6	3.6 ± 1.0	1.9 ± 0.4 *	5.4 ± 0.8
Brain	2.9 ± 0.8	2.6 ± 0.8	3.3 ± 0.5	2.0 ± 0.7
Tumor	3.3 ± 0.3	3.0 ± 0.7	3.2 ± 0.7	3.0 ± 0.8

^1^ Data presented as %ID/g; ^2^ Blocking group (*n* = 4) received SP-141 (1.2 mg) i.p. 30 min before i.v. [^18^F]**1**. * *p* < 0.05 vs. 1 h non-blocking group.

## Data Availability

Data supporting the reported results are available within the article and the Supplementary Materials file.

## Supplementary Materials

The following are available online at https://www.mdpi.com/article/10.3390/ph14040358/s1**,** Figure S1: Surface plasmon resonance analysis of the reference MDM2 inhibitors RG7388 and VIP116, Figure S2: HPLC chromatogram of [^18^F]**1** co-injected with unlabeled 1 for identity confirmation, Figure S3: HPLC chromatogram of [^18^F]**1** in human serum at 3 h after incubation, Figure S4: Effects of Nutlin-3 treatment on the uptake of [^18^F]**1** in MCF-7 and HepG2 cell lines.
