# Supplementary material for: Feasibility of Developing Radiotracers for MDM2: Synthesis and Preliminary Evaluation of an 18F-Labeled Analogue of the MDM2 Inhibitor SP-141
Source: Pharmaceuticals (Basel). 2021 Apr 13;14(4):358. doi: 10.3390/ph14040358 (PMC8070256; doi:10.3390/ph14040358)
Supplement: Supplementary file 1 [file pharmaceuticals-14-00358-s001.pdf]

Supplementary Information

Feasibility of Developing Radiotracers for MDM2: Synthesis and Preliminary Evaluation of an ^{18}F-Labeled Analogue of the MDM2 Inhibitor SP-141

Satish K. Chitneni [1,*], Zhengyuan Zhou [1], Brian E. Watts [2] and Michael R. Zalutsky [1]

[1] Department of Radiology, Duke University Medical Center, Durham, NC 27710, USA; satish.chitneni@duke.edu (S.K.C.); zz89@duke.edu (Z.Z.); michael.zalutsky@duke.edu (M.R.Z.)

[2] Duke Human Vaccine Institute; Duke University, Durham, NC 27708, USA; brian.watts@duke.edu

* Correspondence: satish.chitneni@duke.edu; Tel.: +1-919-684-7809

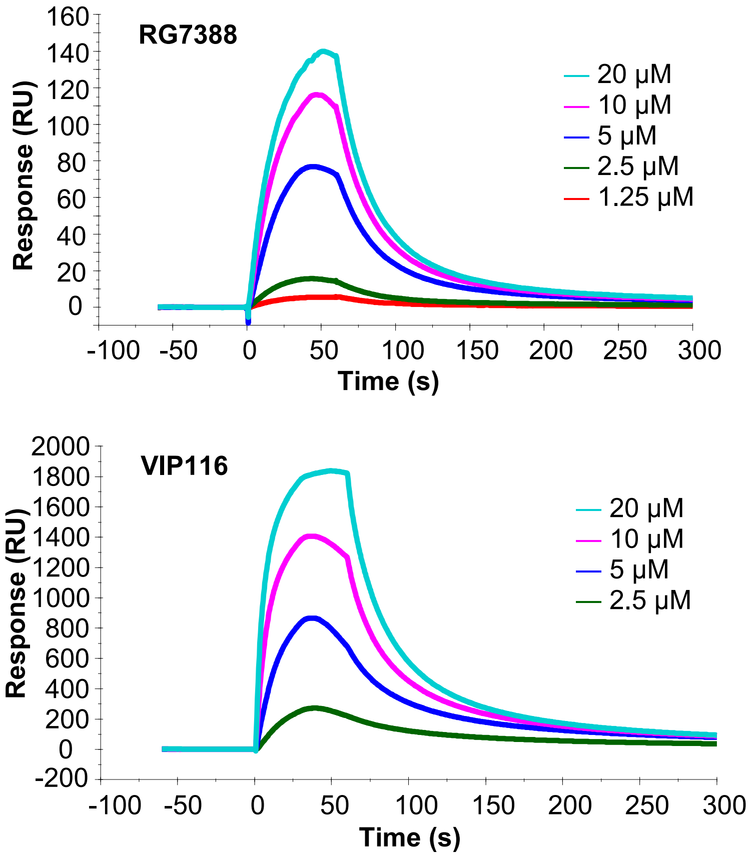

Figure S1. Surface plasmon resonance analysis of the binding interaction between the reference MDM2 inhibitors RG7388 (small molecule) and VIP116 (stapled peptide) with the human recombinant MDM2 protein (Novus Biologicals). Binding responses were recorded at 25°C in the same experiment as SP-141 and the three nonradioactive fluorinated analogues **1**-**3**, as described in the manuscript.

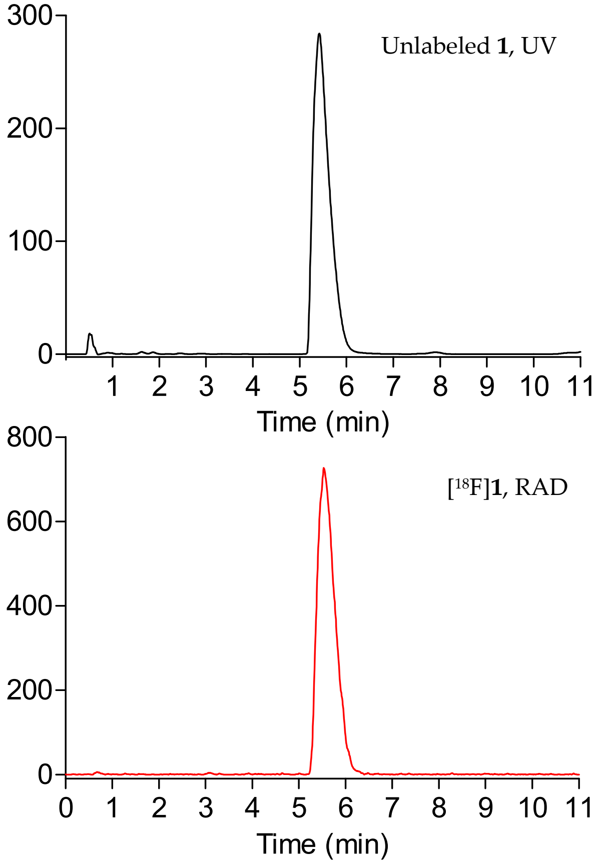

Figure S2. Identity confirmation of [18F]**1** by co-elution with its authenticated nonradioactive analogue **1** by the analytical HPLC system (Knauer, Germany) connected with a XBridge C_{18} column (3.5 μm, 3.0 × 100 mm; Waters) and eluted with acetonitrile and water (45:55; 0.1% TFA) at a flowrate of 1 mL per minute. Retention times: **1** (UV) = 5.4 min, [18F]**1** (RAD) = 5.5 min. Y-axis represents response in millivolts (mV).

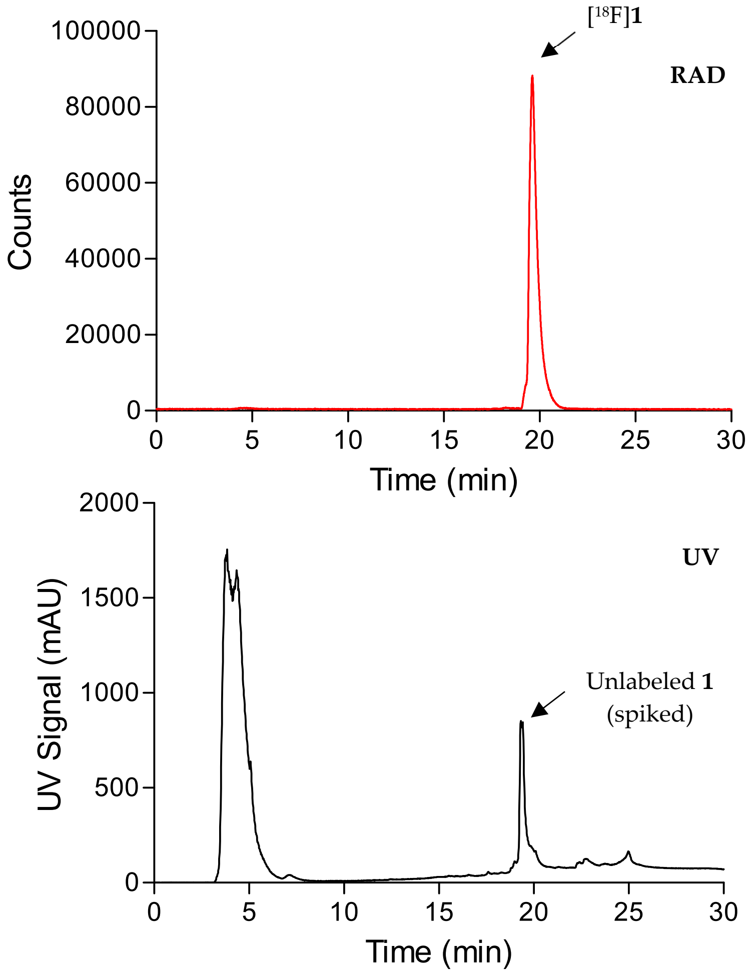

Figure S3. HPLC profile of a human serum sample incubated with [^{18}F]**1** for 3 h. The serum sample was spiked with the authenticated nonradioactive compound **1** prior to HPLC analysis for identity confirmation of the parent [^{18}F]**1** peak. The HPLC system consisted of a Beckman Gold® gradient solvent delivery module connected with a Chromolith® Performance column (4.6 × 100 mm, Millipore Sigma), eluted with gradient mixtures of sodium acetate buffer (0.05 M, pH 5.5) and ethanol as described in the Methods Section.

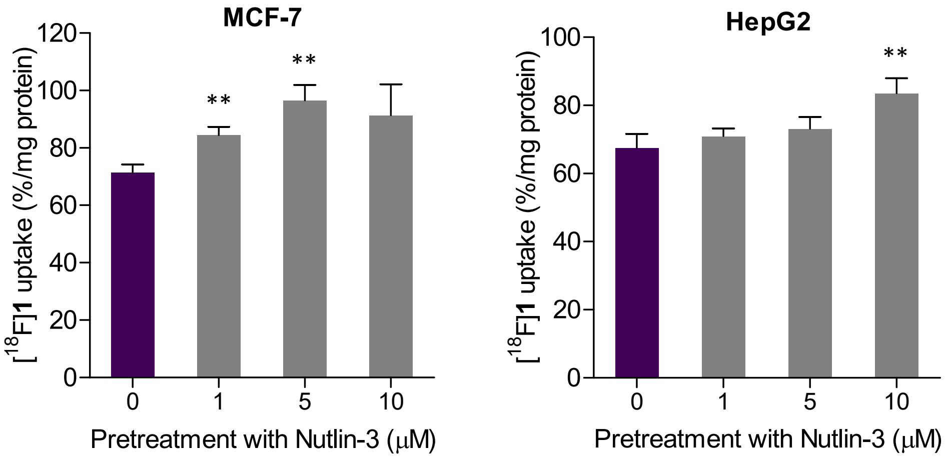

Figure S4. Effect of Nutlin-3 pretreatment on the uptake of [^{18}F]**1** in MCF-7 and HepG2 cells. Cells were treated with varying concentrations of Nutlin-3 (1-10 μM), a well-established MDM2 inhibitor, for 21 h and subsequently evaluated for [^{18}F]**1** uptake for 1 h. **P < 0.01 compared to vehicle-treated control cells (0 μM Nutlin-3).
